# Determinants and effects of academic engagement in university–industry collaboration: a PLS-SEM approach

**DOI:** 10.3389/fpsyg.2026.1745917

**Published:** 2026-04-22

**Authors:** Vladimir Alfonso Ballesteros-Ballesteros, Rodrigo Arturo Zárate-Torres

**Affiliations:** 1 Facultad de Ciencias Económicas, Administrativas y Contables, Fundación Universitaria Los Libertadores, Bogotá D.C., Colombia; 2 Colegio de Estudios Superiores de Administración (CESA), Bogotá D.C., Colombia

**Keywords:** academic engagement, knowledge management, open innovation, PLS-SEM, university–industry collaboration

## Abstract

**Introduction:**

This study examines academic engagement as a mechanism through which universities connect research with societal use. Drawing on a stimulus–organism–response perspective, it investigates the determinants of academic engagement and its effects on knowledge transfer and scientific productivity.

**Methods:**

We administered a cross-sectional survey to 147 full-time faculty members in Colombia using a validated 32-item instrument measured on five-point Likert scales. The model specifies epistemic motivation, instrumental motivation, prior U–I experience, institutional support, and perceived social norms as antecedents; academic engagement as the focal construct; and knowledge transfer and scientific productivity as outcomes. Partial least squares structural equation modelling was used to assess reliability, validity, collinearity, and predictive performance.

**Results:**

The measurement model showed satisfactory psychometric properties, with standardised loadings above 0.70, AVE values ranging from 0.590 to 0.715, composite reliability between 0.852 and 0.899, and a maximum HTMT of 0.829. All antecedents were positively and significantly associated with academic engagement, with institutional support (*β* = 0.373) and epistemic motivation (*β* = 0.320) showing the strongest effects. Academic engagement was positively associated with knowledge transfer (*β* = 0.688) and scientific productivity (*β* = 0.563). The model explained 66.4% of the variance in academic engagement, 47.4% in knowledge transfer, and 31.7% in scientific productivity.

**Discussion:**

The findings position academic engagement as a robust mechanism for translating academic work into external use while sustaining scholarly output. They suggest that universities can strengthen both societal impact and research performance by recognising engagement in workload and promotion systems, reinforcing faculty support structures, and embedding collaboration more systematically into institutional strategy.

## 1 Introduction

Universities are under growing pressure to demonstrate their relevance beyond traditional teaching and research. Governments, firms and civil society organisations increasingly expect higher education institutions to contribute to innovation, competitiveness and social inclusion through direct collaboration with external partners. In this context, interaction between universities and industry has become a key channel for mobilising knowledge, strengthening productive capacities and addressing complex societal challenges (Ballesteros-Ballesteros and Zárate-Torres, 2025a). Recent work suggests that these expectations are unfolding within a changing landscape of science–industry relations marked by new intermediaries, more diverse engagement architectures, and stronger demands for societal relevance, which makes faculty-level collaboration both more strategic and more complex to organise and evaluate (Yström et al., 2025). Faculty members occupy a central position in these dynamics because they control stocks of scientific and technical expertise and decide how to allocate their time between disciplinary work and externally oriented activities. Their involvement in collaborative projects, consulting, specialised training and participation in intersectoral networks can reorient research agendas, reshape academic careers and influence how universities enact their broader social mission. Clarifying how these forms of engagement can be fostered and sustained, and under which conditions they generate benefits rather than costs for academics and institutions, is therefore essential for aligning university strategies with evolving societal expectations (Ahmed et al., 2022).

Prior research conceptualises academic engagement as the active involvement of faculty in collaborative activities, including joint research projects, technical consulting, specialised training and participation in intersectoral networks through which academics work with external partners (Perkmann et al., 2013). This literature distinguishes academic engagement from codified knowledge transfer mechanisms such as patents, licences or spin offs and emphasises its relational and problem-oriented character. Engagement is understood as a way of co-producing knowledge, solving specific problems and strengthening interinstitutional relationships that extend the traditional roles of universities in teaching and research. Empirical contributions show that engagement broadens the range of outputs that matter for impact, highlighting problem-oriented reports, decision support tools, shared data sets and prototypes that inform practice. More recent measurement-oriented research further indicates that engagement should not be treated as a single undifferentiated behaviour. Borlaug et al. (2024), for example, distinguish between research collaboration, consultancy, dissemination, and commercialisation, showing that these modes are associated with partly different determinants. In a related vein, Wang et al. (2025) demonstrate that pathways from knowledge to impact vary across research areas and stakeholder configurations, which reinforces the need for more differentiated and better-specified instruments when analysing faculty interaction with external actors. Together, these insights indicate that academic engagement is not a marginal by-product of university–industry interaction but a central mechanism through which universities seek to create public value and connect research capabilities with the needs of their surrounding environment.

Despite these advances, what we know about the determinants and effects of academic engagement remains fragmented. Existing studies typically examine individual motivations, prior experience with industry, institutional support or perceived social norms in isolation, which obscures how these stimuli jointly shape researchers’ propensity to collaborate with external actors. Evidence on outcomes is similarly partial (Ballesteros-Ballesteros and Zárate-Torres, 2025b). Some findings portray engagement as a vehicle for enriching research agendas and extending societal impact, whereas others fuel concerns that time devoted to external collaboration may divert effort from publishing. Recent instrument-oriented studies begin to address this fragmentation, but they do so in partial and non-cumulative ways. Mascarenhas et al. (2024) separate distinct motivational drivers of university–industry collaboration through multi-item survey measures, while Marques et al. (2024) model university support, perceived benefits, and perceived costs as analytically distinct latent dimensions of collaborative behaviour. Beyond this, scale-development research has started to formalise the psychometric foundations of the field more explicitly. Ortiz-Rojo and Lacruz (2025) validate a professor-centred instrument for university public engagement through confirmatory factor analysis; Verreynne et al. (2025) develop a collaboration-readiness index and diagnostic scale using separate development and confirmation datasets; and Heinen and Bender (2026) measure scientists’ preferences regarding academic engagement directly, thereby extending the field beyond simple frequency-based proxies. At the same time, recent reviews show that university–industry collaboration generates heterogeneous forms of impact and that these are difficult to capture through a single outcome indicator, which strengthens the case for analysing knowledge transfer and scientific productivity as related but distinct consequences of engagement (Cohen et al., 2025). This unresolved tension raises a central puzzle for scholars and decision-makers. Can academic engagement simultaneously foster knowledge transfer to non-academic users and sustain robust scientific productivity, or does it involve systematic trade-offs that vary across institutional contexts? Addressing this question is vital because it goes to the heart of current debates on how universities can deliver impact without eroding their knowledge base and requires an integrated mediational framework (MacKinnon et al., 2007).

This paper addresses this puzzle by examining academic engagement as a mediating mechanism that connects individual and organisational determinants with two core outcomes, knowledge transfer and scientific productivity. We adopt a stimulus–organism–response logic and operationalise it as a mediation model consistent with MacKinnon et al. (2007). More specifically, we conceptualise epistemic motivation, instrumental motivation, prior experience with industry, institutional support and perceived social norms as stimuli that shape researchers’ engagement with external partners. These factors are not treated as a loose list of correlates but as theoretically differentiated dimensions that recent scholarship increasingly measures separately. Epistemic motivation captures the extent to which researchers view collaboration as intellectually generative and aligned with public-science values. Instrumental motivation refers to expected access to funding, data, infrastructure, career advantages and reputational gains. Prior experience with industry captures familiarity with external routines, timelines and languages that can reduce coordination frictions. Institutional support reflects the presence of incentives, administrative facilitation, transfer structures and symbolic legitimation for engagement. Perceived social norms refer to the local climate of peer expectations and the degree to which collaboration with non-academic actors is seen as professionally legitimate. Empirically, we analyse survey data from 147 full-time faculty members in Colombia who are involved in research and applied projects. A 32-item Likert scale instrument, adapted from validated measures, captures these determinants, engagement behaviours and outcomes. Using partial least squares structural equation modelling, we estimate a model that tests the mediating role of academic engagement and examines whether the professors’ h-index moderates the relationship between engagement and knowledge transfer. In doing so, the study advances scholars’ understanding of when and for whom engagement complements rather than crowds out scientific productivity.

The remainder of this article is structured as follows. The next section develops the theoretical foundations of academic engagement, introduces the stimulus–organism–response framework and formulates the hypotheses regarding determinants, mediating mechanisms and outcomes. The subsequent section describes the research design, including the institutional context, sampling strategy, measurement instrument and analytical procedures used to estimate the partial least squares structural equation model. We then present the empirical results, focusing on the relative importance of the determinants, the mediating role of academic engagement and the moderating effect of the h-index on knowledge transfer. The discussion section interprets these findings considering broader debates on university–industry collaboration and outlines their implications for academic careers, institutional policies and public decision-making. The final section concludes by summarising the study’s contributions to theory and practice and by identifying promising directions for future research on the multilevel dynamics of academic engagement.

## 2 Theoretical framework

Academic engagement is a core practice in university–society interaction that is analytically distinct from commercial technology transfer, since it emphasizes collaborative ties rather than appropriation through patents, licenses, or spin-offs. Typical modalities include joint and contract research, specialized consulting, targeted executive training, and active participation in professional and policy networks (Perkmann et al., 2013). These practices enable bidirectional knowledge flows in which faculty act as boundary spanners, translating research insights into socially meaningful applications while channelling external problems back into academic agendas (Perkmann et al., 2021). In this iterative process, knowledge is co-produced, adapted to contextual constraints, and gains public relevance, which strengthens its legitimacy beyond the laboratory or the classroom (Rossi et al., 2017). The resulting outputs tend to be more actionable for firms, governments, and civil society, thereby aligning academic work with institutional missions of innovation, social responsibility, and territorial development (Mascarenhas et al., 2024). Consequently, researchers assume an active role within innovation ecosystems, contributing to the co-construction of high public-value solutions and to the cumulative capacity of organizations to address complex societal challenges in ways that complement, rather than displace, open science and scholarly publication (Scandura and Iammarino, 2022).

Epistemic motivation denotes an internal drive to make sense of complex realities through open and flexible conceptual frameworks, rather than through the pursuit of external rewards such as recognition or financial incentives (Miron-Spektor et al., 2022). This orientation disposes academics to question prior assumptions, search for alternative explanations, and tackle intellectually demanding problems, thereby fostering critical and reflective research practices in higher education settings (Lunn Brownlee et al., 2017). In this context, collaborations with external actors operate not only as channels for applying extant knowledge but also as arenas that broaden researchers’ cognitive horizons by nurturing continuous learning, interdisciplinary openness, and methodological innovation (Franco and Haase, 2015). Consistent with the literature on academic engagement, epistemic motivation underpins boundary-spanning behaviours that enable bidirectional knowledge flows while remaining compatible with open science norms and scholarly publication (Perkmann et al., 2021). Accordingly, epistemic motivation extends beyond compliance with institutional requirements and consolidates as an ethical and epistemological commitment to the public role of academic research. When embedded in contexts of social and technological innovation, it can orient academic cultures toward constructive transformation and the co-production of socially relevant knowledge (Franco and Haase, 2015; Miron-Spektor et al., 2022; Perkmann et al., 2021).

In addition to epistemic drivers, academics engage with external actors for instrumental reasons such as securing funding, accessing professional networks, and using specialized infrastructure that accelerates research and translation (Perkmann et al., 2021). These motives reflect a strategic logic: they help optimize scarce resources, enhance the applicability of findings, and improve the chances that evidence informs organizational and policy decisions (Trouwborst et al., 2024). When aligned with scholarly standards and public value, instrumental motivation complements scientific aims by linking individual ambitions to broader societal purposes and encouraging rigorous, problem-oriented inquiry (Bandhu et al., 2024). In practice, this orientation promotes collaborations where productivity and utility reinforce one another, generating shared value for universities, firms, government agencies, and civil society (Borlaug et al., 2024). Taken together, instrumental considerations can enable researchers to integrate academic interests with collective goals, support co-production with non-academic partners, and translate ideas into context-appropriate solutions, provided that governance arrangements preserve open science norms, maintain transparency around conflicts of interest, and contain transaction costs so that resource acquisition does not crowd out timely dissemination.

Prior experience in non-academic settings strengthens academic engagement by deepening practical skills, expanding professional networks, and sharpening contextual understanding of extra-university demands (Hrivnák and Jarábková, 2022). Such experience equips academics to interpret the languages, timelines, incentives, and coordination routines that characterize intersectoral collaboration, thereby improving expectation management and problem framing with external partners (Woroniecki et al., 2020). As a result, perceived barriers are reduced, more sustainable patterns of articulation are established, and new opportunities for strategic partnerships and joint initiatives emerge across organizational boundaries (Evans et al., 2023). In parallel, external experience can bolster institutional legitimacy, enhance academic visibility, and increase recognition of researchers’ contributions both inside and outside the university, which facilitates access to resources and decision arenas (Fu and Wang, 2024). These dynamics not only support knowledge transfer but also heighten the relevance, applicability, and social impact of research outputs by aligning inquiry with user needs and implementation contexts (Rossi et al., 2024). Taken together, prior extra-academic experience functions as a strategic asset for institutionalizing engagement, informing hiring, promotion, and capacity-building policies that value boundary-spanning trajectories.

The organizational environment plays a central role in promoting academic engagement by shaping the enabling or constraining conditions for university–environment interaction (Perkmann et al., 2021). Clear policies, aligned incentive systems, dedicated transfer units, targeted funding, and fit-for-purpose evaluation frameworks operate as effective mechanisms to normalize and support this practice (Compagnucci and Spigarelli, 2020). These institutional arrangements lower operational barriers, provide procedural guidance, and legitimize engagement as an integral dimension of academic performance alongside publication and teaching (Perkmann et al., 2013). Beyond formal structures, social acceptance also hinges on symbolic endorsement from leaders and peers, whose signals influence perceptions of legitimacy and professional identity (Meyer and Tan, 2025). When universities visibly champion external engagement, they not only enable individual capacity building but also strengthen their social function and institutional reputation through demonstrable contributions to public value (Kitchener et al., 2022). In sum, the organizational environment affects both the feasibility and the recognition of engagement, and thus plays a decisive role in its sustainability, diffusion, and alignment with open science norms and mission-oriented goals.

Shared social norms within academic settings shape academic engagement by signalling what is legitimate, valuable, and expected in research practice (Zhao et al., 2020). These norms emerge through social learning processes that combine peer validation, observation of successful behaviours, and subtle forms of collective pressure that guide everyday decisions about collaboration and outreach (Gelfand et al., 2024). When academic units explicitly encourage interaction with external actors, they nurture organizational cultures that treat engagement as appropriate conduct and weave it into academic identity, promotion criteria, and career narratives (Bogale and Debela, 2024). By contrast, traditional institutional arrangements often reproduce closed models of science that restrict opportunities to interact with societal and industrial stakeholders, narrowing channels for knowledge exchange and limiting the scope of research impact (Medina-Bueno et al., 2024). Taken together, the prevailing normative environment exerts a powerful influence on individual dispositions toward engagement: it shapes perceptions of risk and reward, affects the allocation of time and resources, and determines whether engagement consolidates as a routine and valued dimension of research performance within higher education organizations.

Academic engagement fosters knowledge transfer through push-causation processes in which researchers proactively mobilize expertise to external settings without waiting for explicit demand (De Silva et al., 2021). By contrast with formal instruments such as patents or licenses, informal and relational interactions enable flexible, context-sensitive transfer oriented toward concrete problems; typical modalities include consulting, short targeted training, joint workshops, and participation in professional or civic networks where academics co-develop solutions with non-academic stakeholders (Reed et al., 2021). By expanding boundary-spanning routines and lowering coordination costs, engagement complements rather than substitutes market-mediated mechanisms and positions universities as strategic actors in open innovation and public problem solving (Jonsson et al., 2015). These practices create channels for rapid iteration, mutual learning, and adaptation to local constraints, thereby increasing the likelihood that scientific insights are embedded in deliberative, applied, and sustained processes with measurable social, economic, and environmental effects. This configuration broadens transfer pathways to reach organizations lacking the resources or appropriability conditions for formal technology commercialization and, in parallel, generates feedback loops that refine research questions and heighten the societal salience of academic knowledge.

Although academic engagement primarily targets interaction with external stakeholders, it also yields internal benefits that can strengthen a university’s research system. Collaborations often provide privileged access to empirical settings, proprietary or hard-to-reach data, technical infrastructure, and practitioner expertise, thereby lowering fixed costs of inquiry and opening space for new lines of research to emerge (Marullo et al., 2021; Romero-Sánchez et al., 2024). When aligned with scholarly aims and supported by appropriate incentives, these interactions can translate into peer-reviewed articles, books, and methodological advances with clear domains of application (Gustina et al., 2024). Even so, the conversion of engagement into publications or cumulative scientific capacity is neither automatic nor linear. It depends on institutional support for dedicated research time, robust governance of intellectual contributions, and procedures that formalize results into citable artifacts (Perkmann et al., 2013). Considering this contingent relationship, policies that strategically couple external engagement with research production are essential, including workload models that recognize engagement, funding schemes that require researchable deliverables, and evaluation criteria that credit societally relevant outputs alongside traditional metrics (Nast et al., 2025).

### 2.1 Epistemic motivation and academic engagement

Academic engagement, understood as the active participation of university faculty in collaborative research with external organizations, is now widely recognized as a legitimate mode of university–industry interaction. This engagement includes joint projects, technical consulting, applied knowledge generation, and participation in innovation networks, and it functions as a strategic extension of academic work. Within this framework, epistemic motivation, conceived as the drive to achieve a deep, rigorous, and meaningful understanding of complex problems, emerges as a salient driver of engagement. This intrinsically oriented motivation is expressed in researchers’ interest in exploring novel ideas, addressing real-world challenges, and testing conceptual frameworks in practical contexts. Such a disposition increases scholars’ willingness to participate in boundary-spanning collaboration with non-academic partners (Atta-Owusu and Fitjar, 2022; Miron-Spektor et al., 2022). In turn, interaction with the environment becomes a legitimate source of learning that can inform research agendas, refine methods, and enhance the relevance and visibility of scientific outputs, thereby contributing to the cumulative research capacity of universities while remaining compatible with open science and scholarly publication.

Epistemic motivation denotes researchers’ willingness to invest sustained effort in achieving a precise, nuanced, and context-sensitive understanding of problems, which supports the production of theoretically informed and socially responsive knowledge (Miron-Spektor et al., 2022). From this standpoint, academic engagement arises chiefly from intrinsic motives grounded in the conviction that interaction with external stakeholders enhances the relevance and public value of inquiry, beyond institutional mandates or economic incentives (Sormani et al., 2022). Researchers animated by this orientation are not mere disseminators of results; they purposefully deepen their work through structured dialogue with non-academic actors and iterative exposure to use contexts (Lunn Brownlee et al., 2017). In turn, engagement acquires a genuinely bidirectional character: academic knowledge serves societal needs while real-world challenges feed back into conceptual clarification and methodological refinement within the university (Rossi et al., 2017). Accordingly, engagement rooted in epistemic motivation moves beyond unidirectional transfer and consolidates as a practice of co-creation, broadening the boundaries of scholarly knowledge, strengthening boundary-spanning capabilities, and positioning researchers as proactive agents within innovation ecosystems and processes of social transformation.

The implementation of academic engagement rooted in epistemic motivation depends on individual dispositions and on institutional conditions that structure academic work. Universities that invest in interdisciplinary and applied research tend to create enabling environments for sustained interaction with external stakeholders, legitimizing boundary-crossing practices and reducing coordination frictions (D’Este and Robinson-García, 2023). In settings that safeguard academic freedom and ensure a close thematic fit between industry projects and researchers’ expertise, academics are more willing to collaborate with external partners and to align their scientific agendas with pressing societal challenges; adequate funding and credible partner reputation further strengthen this willingness, whereas short-term orientations weaken it (Clauss et al., 2022). Taken together, these organizational arrangements signal that external collaboration is not merely permissible but integral to producing socially relevant and responsive research. When epistemic motivation is matched with a supportive institutional ecosystem, with clear rules for publication and intellectual property, resources for coordination, and credible recognition, academic engagement is more likely to evolve into a sustained and transformative practice. On this basis, the analytical model advances the following testable proposition:

*H1:* The higher the level of epistemic motivation exhibited by a faculty member, the stronger their engagement in knowledge-driven interactions with external stakeholders.

Building on this proposition, our perspective treats epistemic motivation as a pivotal mechanism that links individual cognition with boundary spanning behaviour. Rather than contrasting curiosity driven academics with more practically oriented colleagues, we emphasise how a strong desire to understand complex problems can be channelled into collaboration with external partners when certain conditions are met. Researchers who are epistemically motivated are more inclined to enter interactions that expose them to unfamiliar contexts, competing interpretations and practical constraints, and to use these encounters to question and refine their own assumptions. At the same time, they are also more likely to be selective about the forms of engagement they pursue, favouring those that allow room for theoretical reflection, methodological rigor and cumulative learning. In our view, the central issue is not whether epistemic motivation supports or hinders engagement, but how it shapes what academics consider meaningful collaboration and how they balance immediate project demands with long term research agendas.

### 2.2 Instrumental motivation and academic engagement

Beyond epistemic motives, faculty may engage with external actors for instrumental reasons linked to concrete operational benefits, such as securing resources, demonstrating impact, or meeting institutional expectations, rather than from an intrinsic commitment to knowledge exchange (Murunga et al., 2024). This orientation does not arise from a desire to understand for its own sake or from an explicit public-service mandate; rather, it reflects the practical utility that collaboration offers for strengthening scholarly work through targeted gains in feasibility and scope (Reiss, 2004). Typical benefits include access to unique datasets that expand the evidentiary base of studies, complementary funding that stabilizes project pipelines, shared infrastructure and technical capabilities that reduce fixed costs, and direct connections to problems and contexts that enrich empirical design and validation (Ozdemir et al., 2023). In this vein, instrumental motivation operates as a strategic logic that treats the external environment as a resource to optimize performance and enhance academic outputs, aligning inquiry with criteria of opportunity, efficiency, and knowledge integration while remaining compatible with open science norms and peer review (Urhahne and Wijnia, 2023).

Researchers whose motivation is predominantly instrumental tend to participate in collaborative arrangements oriented toward concrete results, including contract research, technical consulting, specialized advisory work, and joint developments with external organizations, with the aim of generating applied solutions to partners’ specific needs (Schiefer et al., 2024). These activities are valued not only for their practical impact but also for their capacity to stimulate learning processes and knowledge transfer that enrich academic agendas and strengthen research productivity (Nippa and Reuer, 2019). In institutional contexts that actively promote external interaction, instrumental motivation helps leverage opportunities for innovation, thereby increasing both scientific output and knowledge transfer (Wehn and Montalvo, 2018). In this vein, instrumental motives underpin a utility-oriented form of participation in which academic engagement is sustained by the anticipation of tangible and future benefits (Murayama and von Keyserlingk, 2025). The resulting configuration expands the frontiers of inquiry by aligning projects with real-world constraints and implementation pathways while repositioning academics as boundary spanners across organizational interfaces.

When instrumental motivation is present, researchers’ engagement with external actors becomes more dynamic and sustained, clearly oriented towards the creation of shared value. In this scenario, researchers participate in initiatives where specialised knowledge addresses the concrete needs of organisations, firms and public institutions, while simultaneously strengthening their own research processes and consolidating professional development trajectories. This participation catalyses strategic alliances, applied solution design and collaborative networks that generate visible impacts across academia and the external sector. In doing so, instrumental motivation advances a model of an open, connected university that responds effectively to social, economic and institutional challenges. It not only mobilises individual action but also facilitates collective coordination in cooperation, knowledge transfer and innovation. Based on this theoretical and empirical relationship, the analytical model proposes the following hypothesis:

*H2:* The higher the level of instrumental motivation, the greater the academic engagement of researchers with external stakeholders.

Instrumental motivation therefore invites a more ambivalent view of academic engagement. On the one hand, it can anchor collaboration in concrete projects, timelines and deliverables, which makes interaction with external stakeholders easier to initiate, justify and sustain. From this perspective, engagement becomes part of a deliberate strategy to stabilise funding, expand data access and increase the visibility and applicability of research. On the other hand, a predominantly instrumental orientation may also narrow the scope of collaboration to short term gains, encouraging academics to prioritise projects that yield immediate benefits over those that support deeper learning or long-term agenda building. In such cases, partners may come to be seen mainly as vehicles for resources or indicators of impact, rather than as interlocutors in a shared inquiry process. Our analysis therefore treats instrumental motivation not simply as a positive or negative driver but as a force that shapes the tempo, focus and perceived value of engagement in ways that are open to empirical scrutiny.

### 2.3 Prior industry experience and academic engagement

Prior experience with non-academic actors plays a foundational role in shaping academic engagement (Scandura and Iammarino, 2022). Researchers who have worked with firms, government bodies, or non-profit organizations typically develop communication, negotiation, and project-management competencies that strengthen inter-institutional coordination and boundary-spanning work at later stages (Vega-Jurado et al., 2021). Such trajectories foster sensitivity to the rhythms, languages, and accountability expectations of external environments, reducing perceived risks, uncertainty, and institutional misfit while aligning research agendas with user needs. In parallel, prior engagement expands scholars’ relational capital by embedding them in professional networks that can open opportunities for cooperation, joint problem framing, and access to otherwise hard-to-obtain data or infrastructure (Kronlid and Baraldi, 2020). As a result, extra-academic experience provides not only technical capabilities but also the confidence and situational awareness needed to participate in more complex interaction arenas, consolidating an academic profile that is open to collaboration and better positioned to deliver societally relevant outcomes.

Prior experience operates as a key enabler of academic engagement by helping researchers develop boundary-spanning capabilities and widening their opportunities for interaction across organizational interfaces (Dolmans et al., 2022). Participation in consulting, applied projects, specialized training programs, and outreach events builds practical know-how about how to transfer, adapt, and co-create knowledge in non-academic settings (Compagnucci and Spigarelli, 2024). Rather than diverting effort from scholarship, these learning processes enrich academic profiles and strengthen the capacity to generate impacts beyond the university through problem-oriented collaboration (Engström et al., 2023). At the institutional level, legitimacy accumulated through earlier interactions attracts recognition and eases access to resources and funding while supporting the formation of strategic alliances that sustain longer-term collaboration (Fu and Wang, 2024). In this sense, an accumulated track record with external actors becomes a source of comparative advantage: it improves productivity by accelerating access to data and infrastructure, amplifies impact through implementation pathways, and heightens visibility by embedding researchers in relevant networks, thereby reinforcing ties between universities and their surrounding environments (Sengupta and Rossi, 2023).

Beyond strengthening individual capabilities, prior experience with external actors helps consolidate favourable attitudes toward intersectoral collaboration, thereby nurturing a more open academic culture oriented to exchange and innovation (Li et al., 2023). Researchers who accumulate such trajectories tend to show greater willingness to integrate into trust-based networks, assume leadership responsibilities within project teams, and sustain effective knowledge-transfer processes over time (Bond-Barnard et al., 2018). Evidence also indicates that this behavioural pattern does not emerge spontaneously; rather, it is built gradually through accumulated practice and through positive perceptions of the benefits derived from engagement with the wider environment (Damrich et al., 2022). Consistent with this view, prior industry experience, involvement in commercialization activities, or periods in non-academic roles have been shown to increase the likelihood of academic engagement, underscoring the role of individual antecedents as key determinants of university–environment interaction (Perkmann et al., 2021). Based on this theoretical and empirical relationship, the analytical model proposes the following hypothesis:

*H3:* Researchers with prior experience in activities involving the external sector exhibit higher levels of academic engagement.

Prior industry experience therefore raises important questions about how academics learn to navigate the boundary between university and external organisations. Our perspective treats such experience not merely as a count of past projects, but as a cumulative process through which researchers develop expectations about the value, risks and rhythms of collaboration. Those who have already confronted deadlines, contractual requirements and competing priorities are better equipped to anticipate frictions and to design projects that are both scientifically meaningful and operationally feasible. At the same time, positive experiences can generate a sense of efficacy and professional identity that normalises engagement as part of legitimate academic work, whereas negative encounters may reinforce caution and selective participation. In this view, prior experience does more than increase opportunities for contact. It shapes how academics interpret requests from external partners, how they assess trade-offs between engagement and other commitments, and how they position themselves within emerging collaboration portfolios that link research, teaching and societal impact.

### 2.4 Institutional support and academic engagement

The organisational environment of universities, as expressed in their institutional mission and support policies, plays a decisive role in shaping researchers’ academic engagement (Zhao et al., 2020). Institutional conditions not only delimit opportunities for external collaboration but also mould the individual and collective dispositions of academic staff towards the university’s mission (Philippczyck et al., 2025). In this regard, clear policies, dedicated support units, and recognition mechanisms help legitimise academic engagement as a valued activity within the university mission (Abramo and D’Angelo, 2022). Likewise, infrastructures such as knowledge transfer offices, institutional support programmes, and incentive systems embed university–industry collaboration as a strategic dimension of scholarship, reinforcing legitimacy and value (Yström et al., 2025). Under these conditions, researchers can more easily align their work with external actors, reducing administrative, cultural, and logistical barriers that often constrain such initiatives (Bruneel et al., 2010).

Institutional backing goes beyond the provision of material resources; it encompasses organizational capabilities, infrastructures, and incentive systems that facilitate open and collaborative practices, thereby strengthening innovation and knowledge transfer (Beck et al., 2022). This support also operates as a cultural orientation that communicates clear expectations to researchers and delineates behaviours considered legitimate and appropriate within the university context (Cai and Mountford, 2022). When mentors, academic peers, and institutional structures promote collaborative relationships and offer tangible guidance, early-career researchers perceive greater motivation and confidence to engage in scholarly and transfer activities, especially when workload policies and evaluation criteria explicitly recognize such efforts (Merga and Mason, 2021). Crucially, the perceived legitimacy of this backing acts as a catalyst for academic engagement by reducing professional or reputational risks associated with applied collaboration and by conferring recognition on these practices (Hrivnák and Jarábková, 2022). Consequently, institutional support not only provides resources but also redefines academic engagement within the university ecosystem, signalling that collaboration with external actors is both mission-consistent and professionally valued.

Academic engagement with external actors cannot be understood as the mere outcome of individual motivations; rather, it is a process deeply conditioned by the organizational context in which it unfolds. Factors such as the university’s entrepreneurial mission and the presence of explicit support policies are decisive for activating such engagement (Zhao et al., 2020). Likewise, universities that implement effective support structures, incentives aligned with external collaboration, and appropriate management mechanisms tend to foster partnerships with firms and enhance researchers’ capacity to build sustainable links (Puerta-Sierra et al., 2022). Complementarily, the interaction between responsible leadership and person-organization fit, embedded within a positive institutional culture, strengthens knowledge exchange and facilitates cooperation oriented toward shared objectives (Haider et al., 2022). In consequence, perceived institutional support emerges as an essential enabler of academic engagement: it lowers entry costs, broadens collaboration opportunities, and reinforces researchers’ confidence in their own capabilities while signalling the legitimacy of organizational backing (Perkmann et al., 2021). On this basis, the analytical model advances the following hypothesis:

*H4:* The higher the level of perceived institutional support, the greater the researcher’s academic engagement with external stakeholders.

Taken together, these arguments suggest that institutional support does more than remove practical obstacles; it reshapes how academics interpret and value engagement opportunities. When mission statements, evaluation criteria and everyday mentoring send consistent signals, collaboration with external actors is framed as a legitimate extension of scholarship rather than as a marginal or risky activity. In such environments, researchers can experiment with new partnership formats, knowing that there are routines, offices and people who will help them navigate contracts, intellectual property and expectations from partners. Conversely, ambiguous messages or misaligned incentives may neutralise even strong individual motivations, since academics read them as warnings that engagement may harm their career prospects. Our analysis therefore treats perceived institutional support as a relational construct: what matters is not only what the university formally offers, but whether these arrangements are visible, credible and experienced as fair. This perspective motivates H4 by linking supportive contexts directly to higher and more sustained academic engagement.

### 2.5 Social norms and academic engagement

Beyond formal institutional backing, shared social norms within immediate interaction settings exert a decisive influence on researchers’ willingness to engage in collective activities and to build ties with external actors (Morris et al., 2015; Otten et al., 2024). Departments, research groups, and faculties operate as normative communities that define legitimacy and value in academic practice, steering expectations about what constitutes high-quality research and shaping everyday behavioural cues (Mårtensson et al., 2016). When these units promote external engagement through symbolic recognition, positive weighting in internal evaluations, or role modelling by committed peers, academics come to perceive collaborative participation and knowledge exchange as legitimate and socially expected practices (Joseph and Sengul, 2025). By contrast, where a traditional view centred on conventional scholarly output predominates, engagement with external stakeholders may be interpreted as a deviation from the academic mission or as a loss of institutional focus (Trencher et al., 2014). In this way, the proximate normative environment functions as a behavioural modulator that can either reinforce or inhibit willingness to participate in processes of collaboration and collective problem-solving, with downstream effects on knowledge integration and societal relevance (Osher et al., 2020).

Social norms operate through mechanisms of peer validation, emulation, and pressure, shaping collective dynamics that can trigger sustained change once specific adoption thresholds are reached (Peng and Bai, 2024). In contexts where interaction with external actors is practiced and recognized by leaders and colleagues, researchers perceive symbolic backing that strengthens their commitment and encourages the initiation of new collaborative initiatives (Alpenberg and Scarbrough, 2021). This influence exceeds explicit incentives and manifests in subtle forms of recognition, trust, and prestige among colleagues, which function as effective social incentives (van Houten, 2023). Likewise, an organization’s disposition to share knowledge beyond its own sector is forged in the day-to-day dynamics of university–industry collaboration spaces, where trust, shared norms, and interaction practices are built (Abu Sa’a and Asplund, 2025). Nevertheless, knowledge transfer unfolds on a contested terrain of organizational legitimacy, where offices, departments, and faculties apply *ad hoc* rules, generating internal variation that conditions academics’ willingness to participate in external engagement (Alexander et al., 2020).

When the social norms that shape the academic environment are favourable, entrepreneurial initiatives gain legitimacy and tend to multiply, catalysing new forms of collaboration and prompting transformations in the production and transfer of knowledge (Wadhwani et al., 2017). Such a normative climate supports the coordination of collective efforts, the incorporation of social objectives into the institutional agenda, and the development of cohesive identities oriented towards addressing external challenges (Kozlowski and Ilgen, 2006). Under these conditions, professional identity work is strengthened: values and practices align more readily with external expectations, consolidating an identity that is better integrated with environmental demands (Reissner and Armitage-Chan, 2024). By contrast, where norms do not encourage openness, individuals tend to retreat into conservative routines, restrict their interactions, and avoid initiatives perceived as risky, thereby reducing innovation and resilience; likewise, limited collaboration with external actors constrains access to resources and knowledge that would bolster adaptive capacity (Xia et al., 2024). Within this conceptual framework, the analytical model advances the following hypothesis:

*H5:* The more favourable the social norms within the academic environment towards external engagement, the greater the researcher’s academic engagement.

Social norms thus invite us to treat academic engagement as a collective, rather than purely individual, achievement. What researchers perceive as possible or desirable is filtered through everyday interactions with colleagues, supervisors and leaders who implicitly signal which activities are admired, tolerated or quietly discouraged. In departments where collaboration with external actors is woven into shared stories of good academic work, engagement becomes part of the taken for granted repertoire of how one “does” research and builds a career. Where such norms are absent or ambivalent, the same activities can be read as risky experiments that may jeopardise reputation or advancement. From this perspective, social norms do not simply add another antecedent to the list. They shape how other drivers, such as motivation or institutional support, are interpreted and enacted, and they help explain why similar formal arrangements produce different engagement patterns across units. Our analysis therefore views favourable norms as a crucial mechanism that amplifies and stabilises academic engagement over time.

### 2.6 Academic engagement and knowledge transfer

One of the main recent contributions of the literature has been to recognise academic engagement as a key transmission mechanism, particularly through informal interactions, that functions as an effective channel for knowledge transfer (Perkmann et al., 2021). Unlike traditional mechanisms based on patents, licences, or spin-offs, this form of engagement is expressed in collaborative activities such as joint research projects, technical advisory work, specialised training programmes, and the co-supervision of theses. Such interactions generate impacts that extend beyond the technological and economic domains by delivering social benefits, strengthening student training, and consolidating sustainable university–industry ties (Cohen et al., 2025). Owing to its flexibility, academic engagement allows knowledge to be adapted to specific contexts and to respond to concrete needs, thereby increasing the practical applicability of solutions (De Silva et al., 2021). Consequently, it transcends one-way transfer and operates as a bidirectional process of translation, co-design, and adaptation that embeds academic knowledge within organisational and territorial dynamics, with benefits that go beyond the strictly academic sphere (De Wit-de Vries et al., 2019; Filippetti and Savona, 2017).

Academic engagement, when it complements external needs and is sustained over time, strengthens academics’ capacity to translate disciplinary excellence into effective collaboration and knowledge-transfer processes (Perkmann et al., 2011). Participation in collaborative networks not only mobilises existing knowledge but also enriches it with diverse inputs and practice-based insights that arise from scientific problem-solving, thereby increasing the quality and relevance of results (Kyvik and Reymert, 2017). These dynamics enable co-creation of solutions, shared agenda-setting, and the integration of heterogeneous perspectives throughout the research process, from problem formulation to implementation pathways (Sońta-Drączkowska et al., 2025). Within this framework, the university professor’s role expands from transmitting knowledge to orchestrating boundary-spanning collaboration with external actors, building networks, and managing projects that align academic inquiry with societal demands (Kyvik, 2013). In doing so, well-structured academic engagement positions the university as a central actor in socially oriented innovation, linking research to territorial development and providing a legitimate channel of transfer oriented towards public value and social change (McKelvey and Zaring, 2017).

The relationship between academic engagement and knowledge transfer is mediated by cognitive proximity between researchers and the availability of resources, factors that shape the quality and continuity of interactions and the capacity to sustain knowledge sharing and transformation over time (De Silva et al., 2021). Active participation by academics in institutional knowledge-management practices strengthens ties across departments and among faculty, thereby increasing the university’s ability to share and transmit knowledge more effectively (Vyas, 2024). These connections help translate scholarly insights into contextualised know-how with tangible impact, oriented both to problem solving and to commercialisation pathways (Huang and Xiong, 2023). In turn, universities position themselves as key actors within complex innovation ecosystems, extending their reach and amplifying economic and social impact through boundary-spanning collaboration and implementation channels (Khan et al., 2025). Moreover, engagement generates social impact at the community level and a knowledge impact that enriches professional development by introducing new ideas, skills, and experiential learning cycles (Rossi et al., 2024). Within this conceptual and empirical framework, we advance the following testable proposition:

*H6:* The higher the level of a researcher’s academic engagement, the greater their contribution to the transfer of knowledge to external environments.

From this perspective, academic engagement is not simply an additional channel through which existing knowledge flows, but a context in which knowledge itself is reshaped. When academics work closely with external partners, they confront constraints, tacit practices and evaluative criteria that differ from those of disciplinary communities, and they must translate concepts into forms that can be tested, negotiated and used. These interactions can expose blind spots in prevailing theories, generate new questions and refine methods, so that what is transferred is not a fixed package of results but a living body of ideas that continues to evolve. At the same time, the extent to which engagement strengthens knowledge transfer depends on how far collaborations are structured as joint learning processes rather than one way provision of expert services. Our hypothesis therefore treats academic engagement as a generative mechanism that can transform both the content and trajectories of knowledge as it moves across institutional boundaries.

### 2.7 Academic engagement and research productivity

Although academic engagement is understood as collaboration with external actors, its effects bear directly on research productivity, since it provides a pathway to finance research agendas in which the possibility of publishing is often a key prerequisite for academic participation (Taxt, 2024). Recent evidence indicates that such interactions enrich scholarly work by offering access to field settings in which firms are the object of study, as well as to instruments, datasets, and emergent research topics that would otherwise be difficult to obtain (Kotiranta et al., 2020). In this way, academic engagement strengthens problem-oriented science, deepens theoretical and empirical understanding, and facilitates the application of knowledge in products and services (Yström et al., 2025). In many cases, it catalyses the co-production of knowledge reflected in joint publications, innovative methodologies, and interdisciplinary lines of inquiry, thereby enhancing research with contributions from non-academic partners and increasing the likelihood of producing influential science. Accordingly, academic engagement consolidates as a strategic component of research practice that connects scholarly inquiry to universities’ contemporary social, economic, and technological challenges, aligning funding, access, and research design with credible routes to dissemination and impact (Nast et al., 2025).

Academic engagement broadens faculty members’ professional networks by fostering interactions with external experts and with other researchers oriented toward solving practical problems, thereby strengthening trust and knowledge exchange (Nguyen et al., 2025). Such interactions generate scientific alliances, inter-institutional projects, and co-authorships that enrich research and increase academic visibility through sustainable ties with external stakeholders (Abu Sa’a and Gunnarsson, 2025). At the same time, they facilitate access to funding, research infrastructure, and specialized datasets, strengthening academics’ capacity for rigorous, socially relevant studies and external collaboration (Klincewicz and Zaei, 2025). In applied disciplines such as engineering, health, and the social sciences, academic engagement offers an effective channel for expanding research agendas and strengthening teaching by linking course content to real-world problems. Even in more theoretical areas, it stimulates critical reflection, the reassessment of assumptions, and methodological diversification, thereby reinforcing the academy’s ability to respond to contemporary challenges (Wang et al., 2015). Taken together, academic engagement promotes a fertile interaction between theory and practice that enriches both the research process and scholarly outputs, while consolidating durable collaboration mechanisms across organisational boundaries (Zhuang and Shi, 2024).

The relationship between academic engagement and research productivity is neither automatic nor linear; it depends on factors such as thematic coherence between projects and scholars’ programmes of work, institutional support, and the consolidation of long-term collaborative ties that amplify impacts on academic careers (Plantec et al., 2023). When engagement activities align with established research lines, shared knowledge becomes a direct input for articles, methodological innovations, and applied developments, thereby reinforcing research and transfer in tandem (Hernández-Chea et al., 2021). Institutional recognition, for example through arrangements such as dual affiliation for researchers or company-employed doctoral candidates, legitimises the integration of engagement into academic careers and consolidates it as a sustainable practice (Ljungberg et al., 2025). Likewise, universities that coordinate policies, collaboration networks, and management routines create environments in which academics can expand both their external impact and their scholarly output, strengthening their role within innovation ecosystems (Bürger and Fiates, 2024). In this light, academic engagement is not a deviation from scientific inquiry but, when governed within appropriate institutional frameworks, can extend its relevance, reach, and innovative potential (Muscio et al., 2017).

*H7:* The higher the level of a researcher’s academic engagement, the greater their research productivity.

Taken together, these arguments point to a view of academic engagement and research productivity as mutually shaping elements of a single professional project. When collaborations are thematically aligned with scholars’ core interests, and when institutions provide time, recognition and predictable rules, engagement can help structure research agendas around questions that matter both scientifically and societally. In such cases, external projects do not compete with publication-oriented work but supply data, ideas and partners that expand what researchers are able to do. However, if engagement is added on top of already saturated workloads, or if projects are only loosely connected to existing expertise, the same activities can dilute focus and fragment effort. Our perspective therefore emphasises how academics actively curate their mix of engagements, seeking configurations that sustain their capacity to publish while remaining responsive to external demands. H7 captures this tension by treating engagement as a potential amplifier of productivity whose effects are conditional rather than automatic.

### 2.8 Comparison of effects: knowledge transfer vs. research productivity

Academic engagement is associated with benefits for both knowledge transfer and research productivity, operating as a complement to university research rather than a substitute (Pham et al., 2024). Its effects are heterogeneous and depend on the channel of interaction (consulting, R&D contracts, joint research, or informal ties), the intensity of involvement, and thematic fit with scholars’ programmes of work. Co-production activities such as consulting, collaborative projects, technical assistance, and specialised training strengthen relationships with non-academic actors and can accelerate knowledge circulation and use; however, their performance hinges on project design and start-up, boundary work, contextual anchoring, clear role allocation, and sufficient resources (Zurba et al., 2022). Taken together, engagement functions as a bidirectional mechanism for mobilising knowledge and, when managed at moderate intensity, is often associated with better scientific outcomes than at the extremes, which suggests curvilinear patterns in its relationship with productivity (Perkmann et al., 2021).

Academic engagement also translates and adapts knowledge to non-academic settings through an industrial perspective and the use of boundary objects, which facilitates application and supports applied innovation (Dolmans et al., 2022). Regarding scientific outputs, productivity associated with these interactions depends on thematic continuity and firm support (funding, equipment, and collaboration), which convert resources into time for formalising findings and activate synergies across teams (Zhang J. A. et al., 2024). For innovation performance, the effect of university–industry interaction varies by linkage type: formal R&D alliances reinforce entrepreneurial orientation and speed innovation, whereas very high frequencies of informal contacts can attenuate this effect; the organisation’s developmental stage conditions the magnitude of these relationships (Zhang X. et al., 2024). Overall, effective engagement requires aligning agendas, securing resources, and modulating governance of university–industry ties, combining formal instruments with informal spaces calibrated to organisational maturity.

According to the available scholarly literature, academic engagement is, on average, associated with stronger outcomes in knowledge transfer and with subsequent increases in research productivity, although the magnitude of these effects varies across modes of interaction, disciplines, and institutional contexts. The disciplinary and organisational settings in which engagement occurs also modulate its size. This evidence cautions against unilateral characterisations that portray engagement as more direct, consistent, or immediate in only one domain. Rather, its impact depends on the specific channel, the level of dedicated effort, and the degree of organisational and thematic fit. Reviews further suggest the possibility of diminishing returns when highly applied activities, such as consulting, exceed certain thresholds, which underscores the value of balanced portfolios and careful management of workload and time. Taken together, when mode, intensity, and fit are coherently aligned, academic engagement tends to complement research while enhancing the mobilisation of knowledge, with no systematic evidence of crowding out scientific performance. On this basis, the analytical model advances the following exploratory hypothesis:

*H8:* The effect of academic engagement on knowledge transfer is greater than its effect on research productivity.

Comparing the effects of academic engagement on knowledge transfer and on research productivity invites a more fine-grained view of how collaboration reconfigures academic work. Rather than treating engagement as a uniform driver of outcomes, it is more accurate to see it as a portfolio of practices that redistribute attention, resources and opportunities across these two domains. Engagement that is thematically aligned with existing research and embedded in stable partnerships can simultaneously open channels for the circulation and use of knowledge while generating data, questions and networks that strengthen publication trajectories. By contrast, configurations that are weakly connected to scholars’ core agendas may yield substantial transfer benefits with more modest or delayed impacts on productivity. In our perspective, the relative weight of effects on knowledge transfer and research output reflects how academics and organisations design, sequence and govern their collaborative activities. H8 formalises this intuition by proposing that engagement typically exerts a stronger and more immediate influence on knowledge transfer than on research productivity.

Taken together, the literature reviewed in this section portrays academic engagement as a relational practice shaped by the interplay of individual motivations, prior extra-academic experience, institutional support and local social norms, with differentiated consequences for knowledge transfer and research productivity. However, existing studies typically examine these elements in isolation, focus on single channels of interaction or emphasise either transfer or productivity, which obscures how these drivers operate jointly and through which mechanisms they influence outcomes. In particular, three theoretical issues remain only partially addressed: how epistemic and instrumental motives coexist and interact in shaping engagement, how prior industry experience and perceived institutional support are filtered by normative climates, and whether engagement systematically benefits knowledge transfer more than research output. These unresolved questions delimit a clear space for further theoretical development and motivate the need for an integrated framework that links antecedents, engagement behaviours and dual outcomes within a single model.

Building on this synthesis, we conceptualise academic engagement as a process mechanism that connects individual and organisational determinants with knowledge transfer and research productivity. Rather than treating determinants as a loose list of correlates, we bring them together in a coherent analytical structure that specifies their expected effects on engagement and, through engagement, on both outcome domains. This approach allows us to speak directly to debates on complementarity versus potential crowding out and on the conditions under which engagement strengthens universities’ societal contribution while preserving their research capacity. The next subsection translates this theoretical framing into a research model and a set of hypotheses that can be subjected to empirical scrutiny. It formalises the proposed relationships, clarifies the mediating role of academic engagement and sets the stage for the subsequent description of the methodological design and the PLS-SEM estimation strategy.

### 2.9 Research model and hypotheses development

**Figure 1:**
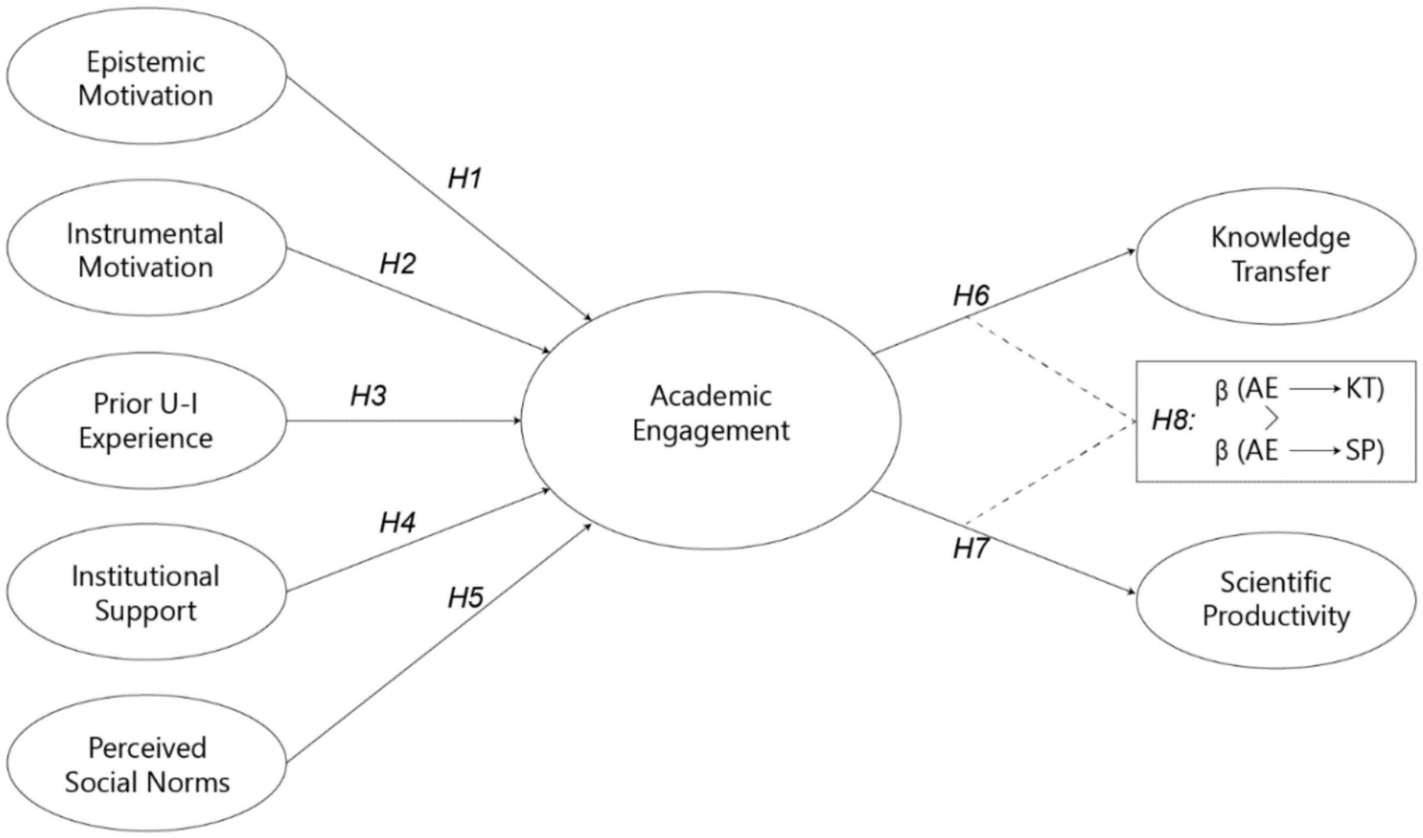
Research model.

This study deepens the analysis of academic engagement as a mechanism of university–environment interaction, focusing on collaborations between academic staff and external institutions. The theoretical model, inspired by Perkmann et al. (2013, 2021), integrates individual determinants (prior research productivity, seniority, relational experience) and institutional determinants (norms, incentives, peer effects) that predispose academics to engage with non-academic actors. These conditions stimulate participation in academic engagement activities, including collaborative research, contract research, consulting, and informal ties, which are modelled here as a mediating variable linking antecedents and outcomes. The evidence indicates that engagement exerts positive effects on two outcomes, namely knowledge transfer and research productivity, while exhibiting recognised heterogeneity across modes, disciplines, and organisational contexts. The model also allows for diminishing returns when applied activities become overly intensive, implying that the relationships between determinants, engagement, and outcomes may be non-linear. Figure 1 summarises the proposed architecture, clarifies the role of the mediator, and guides the formulation of the empirical hypotheses to be tested in subsequent sections.

From a relational cognitive perspective, we specify the stimulus-organism-response model as a mediation framework in which individual and institutional determinants (epistemic and instrumental motivation, prior experience with external actors, institutional support, and perceived norms) act as stimuli; academic engagement operates as the process mechanism; and the observable responses are knowledge transfer and research productivity (MacKinnon et al., 2007). In hypothesis form, each determinant is expected to raise the level of engagement (H1 to H5), and engagement is expected to be positively associated with both outcomes (H6 and H7). We also examine an asymmetry in effect sizes, positing that the impact on knowledge transfer exceeds the impact on research productivity (H8). Mediation will be evaluated by decomposing the total effect into direct and indirect components and by constructing confidence intervals for the indirect effect using bootstrap procedures or the product of coefficients. The analysis will additionally enforce temporal ordering, assess independence of residuals, and verify that no unmodelled interactions confound the mediator–outcome relationship, thereby providing a rigorous test of the proposed process (MacKinnon et al., 2007).

## 3 Method and justification for the PLS-SEM model

This study adopts a quantitative, descriptive approach aimed at empirically testing the proposed relationships between individual and institutional determinants and researchers’ levels of academic engagement. A cross-sectional design was implemented with primary data collected via a survey, enabling the evaluation of the conceptual model’s hypotheses within a single observation window. Academic staff perceptions were measured using polytomous Likert-type scales that are suitable for capturing gradations in epistemic and instrumental motivations, prior experience of interaction with external actors, perceptions of institutional support, and perceived social norms. The instrument operationalises multiple latent constructs and comprises 32 indicators organised into thematic sections, with positively and negatively keyed items to mitigate acquiescence bias. Administration procedures guaranteed anonymity and adhered to ethical standards of informed consent, with clear instructions provided to all respondents. Prior to fieldwork, a pilot test was conducted to refine wording and response times, ensure the clarity and sequencing of instructions, and verify the preliminary internal consistency of the scales. The resulting evidence supports the adequacy of the measurement strategy for testing the study’s hypotheses.

The analysis was conducted using partial least squares structural equation modelling (PLS-SEM), a technique selected for its predictive orientation, its ability to handle data without strict normality assumptions, and its suitability for estimating recursive models that integrate multiple mediations and a large number of items (Hair et al., 2019). The procedure followed two stages. First, the measurement model was assessed using outer loadings, composite reliability, average variance extracted, discriminant validity via the HTMT criterion, and indicator collinearity. To strengthen the psychometric assessment, additional internal-consistency coefficients were also examined, including standardised alpha and McDonald’s omega, while supplementary fit evidence was obtained through SRMR and an auxiliary confirmatory factor analysis estimated for ordinal indicators. Second, the structural model was estimated with standardised coefficients, coefficients of determination, predictive relevance, and effect sizes. Statistical significance was obtained using bootstrapping with a high number of resamples, reporting confidence intervals for direct and indirect effects. Mediation by academic engagement was examined through the magnitude and significance of the proposed indirect effects and their coherence with the causal ordering established by the conceptual model.

### 3.1 Data collection procedure and sample

To empirically evaluate hypotheses H1–H8 described in Section 2, the minimum sample size was determined following Hair et al. (2021), who recommend at least 10 cases for each structural path pointing to a given endogenous construct. In our model, the maximum number of direct influences converges on the endogenous construct of academic engagement, with five incoming paths (H1 to H5). This sets a minimum threshold of 50 observations for adequate model estimation under the 10-cases rule. With 147 valid responses from university researchers, the realised sample comfortably exceeds this requirement and provides sufficient statistical support to test all specified relationships, including H6, H7, and the comparison associated with H8. In addition, the final analytical sample represents 56.1% of the 262 eligible full-time faculty members with active research responsibilities, thereby strengthening the breadth of institutional coverage achieved by the study. Control variables were incorporated to account for observable heterogeneity that could confound the structural paths. This configuration ensures that the evaluation of the hypotheses proceeds with an acceptable level of precision given the model’s complexity and the number of indicators per construct, in line with standard guidance for PLS-SEM sample size adequacy (Hair et al., 2021).

#### 3.1.1 Descriptive data

The questionnaire captured sociodemographic and academic attributes, including sex, age, academic rank, field of knowledge, and years of experience in teaching and research. The sample consisted primarily of assistant and associate professors drawn from several faculties, with most respondents falling between 37 and 52 years of age. As reported in the manuscript, 57% identified as men and 43% as women, and 59% reported more than 2 years of institutional tenure. Disciplinary coverage was broad, including Engineering and Basic Sciences (24%), Economic, Administrative and Accounting Sciences (19%), Human and Social Sciences (18%), Communication Sciences (15%), Psychology (13%), and Law and Political Science (11%). This breadth of professional trajectories and disciplinary homes adds analytical robustness by providing a heterogeneous basis from which to examine the structural relationships specified in the model and by reducing the risk that the results are driven by any single subgroup. Methodologically, such profile variability supports cautious analytic generalisation across the university context rather than confinement to one department or field. In this sense, the composition of the sample is itself an empirical asset because it supplies informative variation that strengthens the interpretation of the observed associations.

#### 3.1.2 Control variables

To isolate the effect of motivations on academic engagement and reduce bias due to structural or demographic heterogeneity, we incorporated control variables associated with institutional location and professional trajectory. In line with the information available in the study, particular attention was paid to home faculty, age, institutional tenure, research experience, and prior collaboration with external actors. The sample spans six faculties with distinct patterns of external interaction: Engineering and Basic Sciences, Economic, Administrative and Accounting Sciences, Communication Sciences, Human and Social Sciences, Law and Political Science, and Psychology. These units differ in research objects, the density of professional networks, and exposure to collaboration opportunities. Controlling for faculty attenuates systematic variation across fields and prevents comparisons from reflecting departmental effects rather than individual dispositions. We also considered age and institutional tenure as covariates to capture the influence of professional trajectory on the formation and use of collaboration networks. Evidence indicates that, with greater experience, researchers expand their pool of collaborators and their access to social capital that facilitates joint projects (Iglič et al., 2017), while longer tenure can strengthen knowledge of procedures and organisational trust (Sarti, 2018). This approach strengthens the internal validity of the analysis and improves the interpretability of the structural estimates.

#### 3.1.3 Sampling

Purposive sampling was employed, appropriate when the target population comprises experts and informants with direct experience of the phenomenon. The accessible population consisted of permanent academic staff with active research duties across the institution’s six faculties. The sampling frame was constructed from official staff lists and records of research projects and research groups, and all eligible individuals were included in the invitation process. Inclusion criteria were holding a full-time or permanent academic appointment, having active research responsibilities during the data collection period, and belonging to one of the institution’s six faculties. Exclusion criteria included adjunct or part-time teaching staff without formal research duties, administrative personnel without academic research responsibilities, and questionnaires that were incomplete or failed the consistency requirements established for the analysis. This strategy ensures relevance and comparability across cases, although it does not imply statistical representativeness; therefore, inferences are analytical. The questionnaire was distributed via institutional email with voluntary participation and anonymity. To reduce coverage bias, reminders were issued and the distribution by faculty and academic rank was monitored. Before full administration, the instrument was also pilot-tested to verify that item wording, sequencing, and instructions were understandable to respondents.

#### 3.1.4 Instrument reliability and validity

Constructs exhibited adequate internal consistency and convergent validity. Composite reliability (ρc) ranged from 0.852 to 0.899, while Cronbach’s alpha ranged from 0.801 to 0.932 when computed at the construct level from the retained indicators. Convergent validity was supported by AVE values between 0.590 and 0.715, and standardised loadings, after dropping one item with loading below 0.70, fell between 0.711 and 0.882. These indicators exceed conventional thresholds (*α* > 0.70; ρc > 0.70; AVE > 0.50; loading > 0.70), confirming the psychometric robustness of the scales employed (Hair et al., 2021). To complement this assessment, additional internal-consistency coefficients were estimated from the item-level dataset. Standardised alpha ranged from 0.801 to 0.932, and McDonald’s omega ranged from 0.804 to 0.932, thereby providing further support for the reliability of the retained constructs. For the full measurement instrument, see Appendix A1. Taken together, these results indicate that the questionnaire provides a robust empirical basis for capturing motivations, institutional conditions, engagement behaviours, and outcome variables within a coherent latent-variable structure.

#### 3.1.5 Sample adequacy

Although the sampling was non-probabilistic, the sample comprises faculty from all six schools of the university, with diverse trajectories in research and external engagement. This multidisciplinary composition and observed heterogeneity provide the variance needed to rigorously test the eight PLS-SEM hypotheses and reflect the institution’s varied university–environment dynamics. In terms of scope, findings are interpreted as an analytic generalisation of the university’s academic ecosystem, without claiming statistical representativeness. In addition, the final analytical sample represents more than half of the eligible population, which strengthens confidence in the breadth of institutional coverage achieved by the study. To reinforce internal validity, the analysis includes controls for research experience, prior collaboration with external actors, home faculty, age, and institutional tenure. These controls mitigate composition and life-cycle biases and enable more precise attribution of estimated effects to the proposed theoretical mechanisms, rather than to individual differences in relational capital or unequal resource endowments across academic units. Accordingly, sample adequacy is supported not only by the minimum-case rule but also by the diversity and institutional reach of the realised sample.

### 3.2 Measurement model assessment

We specified our measurement model with reflective constructs, a standard approach for the variance-based Partial Least Squares Structural Equation Modelling technique. Given that PLS-SEM is robust to non-normal data, we nonetheless assessed the distribution of all indicators. As shown in Appendix A2, the quantile-quantile plots visually confirm the discrete, non-normal nature of our Likert-scale data, thereby validating our methodological choice. Given the five-point Likert scales, analyses of skewness and kurtosis did not reveal problematic departures that would compromise suitability for structural analysis. During measurement model trimming, one indicator with a low loading (item12) was removed. At the item level, all remaining standardized factor loadings comfortably exceeded the 0.70 threshold (minimum = 0.711). Convergent validity was supported by AVE values between 0.590 and 0.715, and composite reliability ranged from 0.852 to 0.899, surpassing standard benchmarks. Discriminant validity assessed via HTMT showed no violations; the maximum HTMT value was 0.829. Taken together, the results support the instrument’s internal consistency and psychometric quality (Table 1).

**Table 1:** Construct reliability and convergent validity.

Latent construct	Abbreviation	Cronbach’s alpha	McDonald’s *ω*	AVE	CR
Epistemic motivation	EM	0.917	0.917	0.682	0.895
Instrumental motivation	IM	0.879	0.881	0.632	0.872
Prior U-I experience	PE	0.801	0.804	0.715	0.883
Institutional support	IS	0.835	0.835	0.692	0.899
Perceived social norms	SN	0.832	0.832	0.679	0.894
Academic engagement	AE	0.932	0.932	0.675	0.892
Knowledge transfer	KT	0.895	0.895	0.646	0.879
Scientific productivity	SP	0.858	0.859	0.590	0.852

Discriminant validity was assessed using the Heterotrait–Monotrait ratio, a robust criterion for PLS-SEM. All pairwise construct correlations were below the conservative 0.90 threshold, indicating adequate conceptual separation among the latent variables. The HTMT between AE and KT was 0.829, close to the stricter 0.85 cutoff yet still below it, thus supporting the discriminant validity of these constructs. The literature also accepts a more lenient 0.90 threshold, particularly in complex models with multiple constructs and paths, provided there is solid theoretical grounding and internal consistency in measurement (Henseler et al., 2015). In this study, the conceptual proximity of AE and KT, given that academic engagement functions as an intermediate mechanism in knowledge transfer, is consistent with a moderate association. To further strengthen the assessment of measurement adequacy, a supplementary confirmatory factor analysis was estimated using a diagonally weighted least squares specification appropriate for ordinal indicators. This auxiliary analysis showed excellent fit, with NNFI/TLI above 0.99, GFI = 0.974, and SRMR = 0.052. Taken together, the evidence supports adequate discriminant validity and the overall adequacy of the reflective measurement specification.

### 3.3 Structural model evaluation

Structural model evaluation followed Hair et al. (2019), considering path significance, explained variance (*R*^2^) of endogenous constructs, effect sizes (*f*^2^), and collinearity (VIF). All paths were statistically significant (*p* < 0.001, two-tailed). Among the predictors of Academic Engagement, the largest positive effect came from Institutional Support (*β* = 0.373), followed by Epistemic Motivation (*β* = 0.320), Perceived Social Norms (*β* = 0.253), Prior U-I Experience (*β* = 0.235), and Instrumental Motivation (*β* = 0.159). In turn, Academic Engagement strongly affected Knowledge Transfer (*β* = 0.688) and had a moderate to large effect on Scientific Productivity (*β* = 0.563) (Table 2). Endogenous constructs showed *R*^2^ of 0.664 for AE, 0.474 for KT, and 0.317 for SP, indicating moderate to substantial explanatory power. Effect sizes ranged from small, for example IM → AE (*f*^2^ = 0.065), to large, for example AE → KT (*f*^2^ = 0.900) and AE → SP (*f*^2^ = 0.465). VIF values were comfortably below 3, ruling out multicollinearity. Moreover, 95% bootstrap confidence intervals based on 5,000 resamples excluded zero for all paths, reinforcing the robustness of the hypothesised relationships.

**Table 2:** Path coefficients and hypothesis testing results.

Hypothesized path	Expected effect	*β* (Standardized)	Standard Error	*t*-value	Decision
EM → AE	Higher EM increases AE	0.320	0.056	5.670	Supported
IM → AE	Higher IM increases AE	0.159	0.052	3.059	Supported
PE → AE	Higher PE increases AE	0.235	0.053	4.445	Supported
IS → AE	Higher IS increases AE	0.373	0.055	6.776	Supported
SN → AE	More favourable SN increases AE	0.253	0.045	5.598	Supported
AE → KT	Higher AE increases KT	0.688	0.050	13.822	Supported
AE → SP	Higher AE increases SP	0.563	0.056	10.124	Supported

All coefficients are standardized. Bootstrap sample = 5,000. Hypotheses were supported at *p* < 0.05 (one-tailed test).

Structural model evaluation showed substantive explained variance. Academic Engagement had *R*^2^ = 0.664, Knowledge Transfer had *R*^2^ = 0.474, and Scientific Productivity had *R*^2^ = 0.317, indicating moderate to high predictive power. Regarding effect sizes, the impact of AE on KT was large (*f*^2^ = 0.900) and on SP was medium to large (*f*^2^ = 0.465). Paths into AE were small to medium in magnitude, ranging from 0.065 to 0.351. Collinearity indices were approximately 1.07–1.19 for the AE block and 1.00 when a path had a single predictor, ruling out multicollinearity. Because the proposition concerning the stronger effect of academic engagement on knowledge transfer than on scientific productivity is, substantively, a comparative asymmetry claim, it is interpreted from the relative magnitude of these two bootstrapped coefficients. On that basis, academic engagement exhibits a clearly stronger association with knowledge transfer than with scientific productivity. Positive *Q*^2^ values for all endogenous constructs further support the predictive relevance of the model (Table 3).

**Table 3:** Summary of structural model assessment results.

Hypothesized path	*f* ^2^	*R* ^2^	*Q* ^2^	VIF
EM → AE	0.274	0.664	0.377	1.114
IM → AE	0.065	0.664	0.377	1.145
PE → AE	0.139	0.664	0.377	1.185
IS → AE	0.351	0.664	0.377	1.183
SN → AE	0.178	0.664	0.377	1.072
AE → KT	0.900	0.474	0.197	1.000
AE → SP	0.465	0.317	0.132	1.000

*f*^2^ indicates the effect size of each predictor on its dependent construct; thresholds of 0.02, 0.15, and 0.35 denote small, medium, and large effects, respectively (Cohen, 1992). *R*^2^ denotes the proportion of variance explained in each endogenous construct. *Q*^2^, obtained via k-fold cross-validated redundancy, assesses predictive relevance; values greater than zero indicate adequate relevance. VIF (variance inflation factor) values below 5 suggest the absence of problematic multicollinearity. Results are based on PLS-SEM with 5,000 bootstrap resamples.

**Figure 2:**
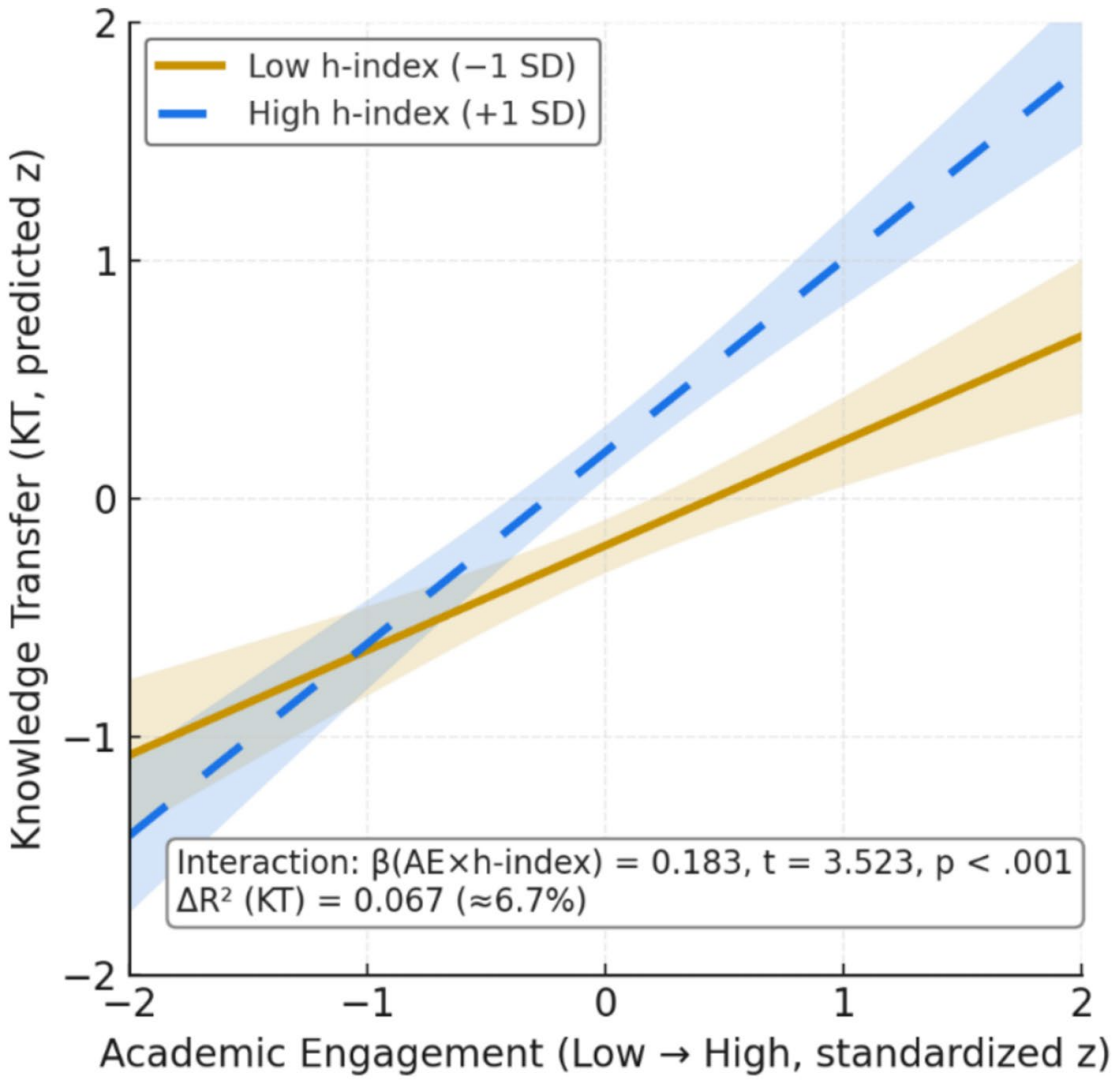
Moderating effect of the *h*-index on the relationship between academic engagement and knowledge transfer. Two-way interaction with continuous moderator; ribbons show 95% CIs using bootstrap SEs (no covariance).

Adding the interaction between the h-index and academic engagement (AE) on knowledge transfer (KT) increased KT’s *R*^2^ from 0.474 to 0.541 (Δ*R*^2^ = 0.067), representing a 6.7% gain in explained variance. The interaction effect was positive and statistically significant (*β* = 0.183, *p* < 0.001; 95% CI [0.087, 0.282]; Table 4), supporting H8 by indicating that the h-index positively moderates the AE → KT relationship. The direct effect of the h-index on KT was also positive and significant (*β* = 0.197, *p* < 0.001). To visualise the moderation, simple slopes were estimated following a two-way approach (Dawson, 2014). Figure 2 shows that the AE → KT relationship remains positive across levels of the h-index and becomes steeper at higher levels of scholarly standing. Overall, these results suggest that prior scholarly standing may strengthen the extent to which academic engagement translates into knowledge transfer outcomes, particularly in university–industry collaboration settings (Perkmann et al., 2013; Scandura and Iammarino, 2022; Mascarenhas et al., 2024).

**Table 4:** Testing hypothesis for moderating effect.

Predictors	Main effect std. beta	Interaction effect std. beta	*t*-value
AE	0.623	–	11.219
h-index	0.197	–	3.491
h-index × AE	–	0.183	3.523
*R* ^2^	0.541	–	–
*R*^2^ change (Δ*R*^2^)	0.067	–	–

Bootstrapping results (*n* = 5,000; one-tailed). All reported effects are statistically significant at *p* < 0.001. *R*^2^ denotes variance explained in Knowledge Transfer; Δ*R*^2^ is the increase in *R*^2^ after adding the interaction term (AE × h-index).

## 4 Discussion

Academic engagement (AE) emerges from our results as a bridge between the university and its environment, activated jointly by individual and institutional conditions. Institutional support is the strongest predictor of AE (*β* = 0.373), while epistemic motivation remains a robust determinant (*β* = 0.320), indicating that researchers oriented toward understanding and applying knowledge are more likely to collaborate with non-academic actors. This configuration suggests that engagement is not an external add-on to science, but a consistent expression of inquiry when academics confront demanding and socially relevant problems. Even after accounting for institutional support and perceived social norms, intrinsic drivers retain substantial explanatory power. In this respect, our findings are consistent with evidence showing that researchers’ societal and industrial engagement is shaped by a combination of motivations, career trajectories, and organisational conditions rather than by any single determinant (Borlaug et al., 2024; Mascarenhas et al., 2024). They also extend this literature by quantifying the relative contribution of these drivers in a teaching-oriented, resource-constrained Latin American university, a setting still underrepresented in the field.

The positive and statistically significant effect of instrumental motivation on engagement with external actors (IM → AE: *β* = 0.159, *p* < 0.01) complements this picture and points to the coexistence of distinct rationales in collaboration decisions. Faculty not only seek to understand complex problems, they also pursue access to funding, networks, data, infrastructure, and reputational opportunities that can expand research capacity and accelerate project execution. This combination of cognitive interest and opportunity-seeking is sometimes portrayed as a source of tension, yet our results suggest that it may instead operate as a functional balance that facilitates collaboration. In contexts where engagement opportunities are unevenly distributed, as is often the case in teaching-oriented universities in Latin America, instrumental motives may play a particularly important mobilising role because they lower the threshold for initial participation. Similar patterns have been identified in cross-border European regions, where expected gains in resources, visibility, and commercial opportunities coexist with non-pecuniary motives in shaping collaboration decisions (Mascarenhas et al., 2024). Our findings therefore challenge simple dichotomies between intrinsic and extrinsic drivers and instead support a view of AE as a layered, context-dependent practice.

Accumulated experience with external actors is also positively and significantly associated with faculty engagement (PE → AE: *β* = 0.235, *p* < 0.001). Direct contact with organisations provides practical learning, reduces uncertainty, increases self-confidence, and expands relational capital, which in turn facilitates subsequent interactions. Each collaboration can lower coordination costs and create a cumulative process in which engagement becomes progressively easier and more meaningful. In our study, this pattern is especially relevant because the sample comes from a primarily teaching-oriented institution that conducts applied research in selected fields, which suggests that even limited experience may meaningfully increase willingness to collaborate. This refines earlier work focused mainly on highly research-intensive universities by showing that boundary-spanning trajectories can also develop in more modest settings. It also resonates with recent evidence showing that collaboration readiness, familiarity with routines, and prior contact with external organisations are important for sustaining university-business collaboration over time (Verreynne et al., 2025). From a management perspective, these findings support the creation of low-risk entry opportunities such as pilot projects, short consultancies, and micro-contracts that allow researchers to build collaboration capabilities incrementally.

Institutional support emerges as a key facilitator of engagement with external actors, with a positive and statistically significant association with AE (IS → AE: *β* = 0.373, *p* < 0.001). Perceived technical, regulatory, and symbolic backing is linked to greater faculty participation even where knowledge transfer capabilities are still developing. Early and concrete organisational signals, such as internal guidelines, standard contract templates, legal advice, targeted funding calls, and modest incentives, can help trigger participation and sustain it over time (Al-Taie and Khattak, 2024). Our findings are broadly compatible with studies emphasising the importance of organisational arrangements, but they also add precision by documenting how strongly support matters relative to individual motivations (Fan et al., 2022). This interpretation is consistent with recent work showing that university-industry collaboration is shaped by social exchange dynamics and by institutional arrangements that reduce uncertainty and transaction costs for academics and external partners alike (Marques et al., 2024). It also aligns with broader arguments that changing forms of science-industry relations require more deliberate institutional architectures to organise engagement, especially where collaboration is not yet fully routinised (Yström et al., 2025). Under these conditions, engagement becomes part of ordinary academic work rather than an exceptional activity undertaken by only a few faculty members.

Perceived social norms within academic settings are another robust and policy-relevant predictor of engagement with external actors (SN → AE: *β* = 0.253, *p* < 0.001). Shared expectations, visible role models, and local routines shape willingness to collaborate and can trigger peer imitation that lowers the social and professional cost of first interactions. When departments, research groups, and colleagues openly legitimise engagement, approval cues intensify, sustaining ongoing initiatives and attracting new participants across units. Our results therefore extend previous accounts of organisational culture by showing that normative climates do not simply accompany engagement, but actively structure who participates, in what ways, and with what persistence. This interpretation is consistent with cross-country evidence showing that researchers engage with society through heterogeneous channels and that these patterns are partly embedded in organisational and disciplinary environments (Borlaug et al., 2024). It also suggests that formal policies and incentives will be less effective if they are not accompanied by local cultures that render collaboration professionally legitimate. Universities seeking to scale academic engagement should therefore complement central support mechanisms with culture-building initiatives at the level of departments and research groups.

Regarding outcomes, AE shows positive effects on both knowledge transfer and scientific productivity, although with different magnitudes and temporalities. The effect on knowledge transfer is stronger and more direct (AE → KT: *β* = 0.688; *R*^2^ = 0.474), which is consistent with the applied orientation of collaborative projects, consulting, and technical assistance. The effect on scientific productivity is also positive, although more moderate (AE → SP: *β* = 0.563; *R*^2^ = 0.317), suggesting that converting engagement into articles and other formal outputs requires thematic alignment, time, and additional organisational support. These results are consistent with studies portraying engagement as a problem-oriented practice, yet they challenge narratives that assume an inevitable trade-off between collaboration and publication. In our setting, engagement appears broadly complementary to research, although the pathways and time horizons differ across outcomes. This conclusion aligns with recent evidence showing that academic engagement can positively influence scientific impact when interactions are coherent with researchers’ agendas and supported by appropriate collaboration structures (Nast et al., 2025). It is also consistent with findings indicating that publicly co-funded industry-science collaboration can strengthen scientific production rather than crowd it out, particularly when collaboration is institutionally scaffolded (Hünermund et al., 2026).

The moderation analysis adds a further layer to this interpretation. Adding the interaction between the h-index and AE on KT increased KT’s *R*^2^ from 0.474 to 0.541 (Δ*R*^2^ = 0.067), and the interaction effect was positive and statistically significant (*β* = 0.183, *p* < 0.001; 95% CI [0.087, 0.282]; Table 4). The direct effect of the h-index on KT was also positive (*β* = 0.197, *p* < 0.001). Simple slopes estimated using a two-way approach showed that the AE → KT relationship remains positive across levels of the h-index and becomes steeper at higher levels of scholarly standing (Dawson, 2014). Taken together, these findings suggest that prior scientific reputation may operate as a resource that amplifies the returns to academic engagement, particularly in university-industry collaborations, by strengthening researcher credibility and fostering greater trust, openness, and willingness among external partners (Perkmann et al., 2013; Scandura and Iammarino, 2022; Mascarenhas et al., 2024). This result should nonetheless be interpreted as a supplementary conditioning mechanism rather than as the primary basis for the comparative asymmetry between AE → KT and AE → SP.

Taken together, these findings support a validated model that contributes both theoretically and empirically to the study of AE as a relational mechanism linking universities with their environment. The model shows how motivational, experiential, and contextual factors jointly shape faculty behaviour and how that behaviour translates into differentiated outcomes in knowledge transfer and scientific productivity. By integrating these elements within a single analytical specification, the study advances beyond prior work that has often examined determinants or outcomes in isolation. Evidence from a Latin American, teaching-oriented university also broadens the empirical base of the literature, which remains heavily concentrated in research-intensive institutions located in mature innovation systems. This contextual extension is not merely descriptive. It suggests that meaningful engagement can become structurally embedded even in resource-constrained settings when institutional support, social norms, and individual motivations align. In this sense, the study complements recent calls to broaden the geographic and organisational scope of research on science-industry relations and to recognise the diversity of pathways through which engagement is organised and sustained across systems (Yström et al., 2025; Wang et al., 2025).

The article also contributes conceptually by specifying how epistemic and instrumental motivations, prior industry experience, institutional support, and social norms can be integrated within a unified stimulus-organism-response framework in which AE functions as a mediating process rather than as a simple count of activities. This perspective helps explain how individual and organisational drivers operate jointly and how they become consequential for dual outcome domains. By modelling the effects of AE on both knowledge transfer and scientific productivity within the same framework, the study clarifies the conditions under which complementarities arise and directly addresses long-standing concerns about the possible crowding out of research. In doing so, it refines existing multilevel accounts of engagement and offers an analytical template that may travel beyond highly resourced universities. The broader implication is that engagement should not be conceptualised solely as a peripheral third-mission activity, but as an organisationally conditioned process through which academic work can be made simultaneously more outward-facing and more cumulative in scientific terms.

The findings carry several implications for university management and public policy. First, they underscore the importance of strengthening the epistemic core of academic work while simultaneously providing accessible procedural, legal, and administrative support for collaboration. Policies that recognise engagement in workload models, promotion criteria, and internal evaluation systems are likely to encourage more systematic and thematically coherent interaction with external actors. Second, the results highlight the value of culture-building initiatives at the level of departments and research groups, where social norms, peer recognition, and role models can normalise collaboration and reduce perceived risks. Third, universities and funding agencies should adopt measurement systems that capture multiple forms of impact, combining traditional indicators of scientific production with evidence on knowledge use, adoption, and utility. Such integrated portfolios would allow institutions to align incentives with mission-oriented objectives and to manage engagement as a strategic component of their contribution to innovation and social development. These recommendations are broadly compatible with recent work emphasising collaboration readiness, diversified impact pathways, and the need for more nuanced institutional metrics in university-business collaboration (Verreynne et al., 2025; Wang et al., 2025).

Finally, the study has limitations that invite cautious interpretation and point to several avenues for future research. The analysis is confined to a single institution and relies on cross-sectional, self-reported data, which restricts the strength of causal inferences and the generalisability of effect sizes across contexts. Future work should therefore extend the framework through longitudinal designs that track trajectories of engagement and impact over time, comparative studies across institutional types and national settings, and mixed-method approaches that connect survey evidence with qualitative analysis of project governance, intellectual property arrangements, and data practices. It would also be valuable to incorporate additional indicators of knowledge transfer and scientific productivity and to examine possible non-linearities or alternative mediating and moderating mechanisms. Despite these limitations, the study offers a coherent and empirically grounded model that can guide subsequent investigations and inform policy debates on how universities can organise engagement in ways that support both societal impact and robust scholarship.

## 5 Conclusion and limitations

This study examined academic engagement as a mediating mechanism linking individual and organisational determinants with two core outcomes, knowledge transfer and scientific productivity, in a university setting characterised by teaching intensity, applied research activity, and relative resource constraints. Our findings suggest, first, that academic engagement is best understood not as a peripheral or episodic activity, but as a structured relational process shaped by the joint influence of epistemic motivation, instrumental motivation, prior experience with external actors, institutional support, and perceived social norms. In that respect, the article moves beyond approaches that consider these factors separately and instead shows how they combine to shape faculty involvement with non-academic partners. More broadly, the results indicate that academic engagement constitutes a relevant organisational channel through which universities connect scholarly work with external problems, users, and contexts of application. This is important because it shifts attention from engagement as an outcome in itself to engagement as a mechanism through which universities organise their societal interface.

Second, the study contributes to ongoing debates on whether engagement with external actors complements or displaces conventional academic activity. The results suggest that academic engagement is positively associated with both knowledge transfer and scientific productivity, although with different strengths and likely different temporalities. Its association with knowledge transfer is stronger and more immediate, which is consistent with the applied orientation of collaborative projects, consulting, and technical interaction. Its association with scientific productivity is also positive, although more moderate, suggesting that the translation of engagement into publications and other codified outputs depends on additional conditions such as thematic continuity, resource availability, and the stability of collaborative arrangements. In this sense, our findings do not support a simple zero-sum view of university engagement. Rather, they indicate that the relationship is better understood as one of conditional complementarity: engagement can reinforce both external use and scholarly accumulation when it is aligned with researchers’ programmes of work and supported by credible institutional arrangements.

Third, the article makes a methodological contribution by showing that the proposed analytical model is not only conceptually coherent but also empirically robust. The measurement model displayed satisfactory reliability and convergent validity, with retained indicators showing adequate loadings, average variance extracted above conventional thresholds, and composite reliability values consistent with strong internal consistency. Additional evidence from Cronbach’s alpha and McDonald’s omega reinforced this assessment, while HTMT results supported discriminant validity among the constructs. At the structural level, the model showed substantial explanatory power for academic engagement, moderate to substantial explained variance for knowledge transfer and scientific productivity, low collinearity, and positive predictive relevance. These results matter because they indicate that the article’s substantive claims rest not only on theoretical plausibility, but also on a model with acceptable validity, stability, and predictive performance. The study therefore contributes to the literature not only by refining theoretical arguments, but also by offering a rigorous empirical specification of academic engagement and its differentiated outcomes.

Fourth, the article refines existing multilevel accounts of university-industry collaboration by specifying how motivational, experiential, and contextual factors combine to shape academic engagement and, through engagement, influence distinct outcome domains. The stimulus-organism-response perspective proved useful because it allowed engagement to be treated as a mediating process rather than as a simple count of collaborative activities. By modelling knowledge transfer and scientific productivity within the same framework, the study responds directly to the question that motivated the analysis at the outset: whether universities can strengthen their societal contribution without undermining their research capacity. Evidence from a Latin American, teaching-oriented university adds further value by extending the empirical conversation beyond mature, research-intensive systems, where much of the literature remains concentrated. The contribution is thus not merely contextual. It also lies in showing that meaningful engagement can emerge under more constrained conditions when institutional support, prior experience, social norms, and individual motivations are aligned in ways that make collaboration both feasible and professionally legitimate.

Finally, the findings carry several implications for university management and public policy. They suggest, first, that institutions should strengthen the epistemic core of academic work while simultaneously providing accessible procedural, legal, and administrative support for collaboration. Second, they indicate that engagement is more likely to become systematic when recognised in workload allocation, promotion criteria, and internal evaluation systems (Zhang et al., 2025). Third, they highlight the importance of local academic cultures, since social norms, peer recognition, and visible role models help normalise collaboration and reduce perceived risks. At the same time, these implications should be interpreted in light of the study’s limits (Yu et al., 2025). The analysis is confined to a single institution and relies on cross-sectional, self-reported data, which constrains causal inference and limits the broader generalisability of effect sizes. Future research should therefore extend this framework through longitudinal, comparative, and mixed-method designs that can better capture how engagement evolves and under what conditions it produces sustained scholarly and societal returns.

## Data Availability

The raw data supporting the conclusions of this article will be made available by the authors without undue reservation.
