# Supplementary material for: Determinants and effects of academic engagement in university–industry collaboration: a PLS-SEM approach
Source: Front Psychol. 2026 Apr 22;17:1745917. doi: 10.3389/fpsyg.2026.1745917 (PMC13143539; doi:10.3389/fpsyg.2026.1745917)
Supplement: Supplementary file 1 [file Supplementary_file_1.docx]

**Appendix A1**

**Table A1.** Constructs, item codes, and survey statements (Likert 1–5).

| **Item Code** | **Item Statement** |
| --- | --- |
| *Construct: EM* | *Epistemic motivation* |
| EM1 | Collaborating with external organizations embodies core public science values. |
| EM2 | Working with non-academic partners stimulates new research questions. |
| EM3 | Knowledge exchange with practitioners improves the external validity of my theoretical frameworks. |
| EM4 | [R] Collaborating with external actors distracts me from important scientific problems. |
| *Construct: IM* | *Instrumental motivation* |
| IM1 | I collaborate to access data, materials, or equipment not otherwise available. |
| IM2 | I consider the career and reputational benefits of collaborating with external partners. |
| IM3 | External funding (e.g., contract research, consulting) influences my decision to collaborate. |
| IM4 | [R] Collaborating with external partners provides no practical advantages for my projects. |
| *Construct: PE* | *Prior U–I Experience* |
| PE1 | I have conducted collaborative research with non-academic organizations. |
| PE2 | I have carried out contract research funded by external partners. |
| PE3 | I have provided technical consulting to external organizations. |
| PE4 | [R] I have avoided informal interactions (ad hoc advice, networking) with practitioners. |
| *Construct: IS* | *Institutional Support (perceived)* |
| IS1 | My university provides standard contracts and clear guidelines for external collaboration. |
| IS2 | Interdisciplinary structures and technology/knowledge transfer offices facilitate collaboration. |
| IS3 | I receive administrative support for agreements, intellectual property (IP), and compliance. |
| IS4 | [R] Internal policies hinder collaboration with external organizations. |
| *Construct: SN* | *Perceived Social Norms* |
| SN1 | In my department, collaboration with external organizations is valued. |
| SN2 | Role models who collaborate with industry encourage my participation. |
| SN3 | Co-authors and peers influence my willingness to collaborate with external partners. |
| SN4 | [R] Collaborating with external actors is viewed as outside my field’s academic identity. |
| *Construct: RC* | *Recent Collaboration (past 12 months)* |
| RC1 | I engaged in collaborative research with companies, NGOs, or public agencies. |
| RC2 | I undertook contract research with external partners. |
| RC3 | I provided consulting or technical advice to external organizations. |
| RC4 | [R] I avoided informal interactions aimed at knowledge exchange. |
| *Construct: KT* | *Knowledge Transfer Outcomes (past 12 months)* |
| KT1 | External partners used results or ideas that emerged from our interactions. |
| KT2 | Collaborations produced applied outputs (e.g., prototypes, pilots, actionable reports). |
| KT3 | Partners reported that the knowledge exchanged was useful for their activities. |
| KT4 | [R] Collaborations yielded little beyond routine IP or administrative processes. |
| *Construct: SP* | *Scientific Productivity (past 12 months)* |
| SP1 | Collaborations with external partners led to follow-on publications. |
| SP2 | These collaborations improved my ability to secure competitive public grants. |
| SP3 | Collaboration with external organizations increased my publication output. |
| SP4 | [R] Collaborating with external partners reduced my opportunities for co-authored publications. |

Notes: [R] = reverse-coded. Reference period for RC, KT, and SP: past 12 months.
