# Supplementary figures and images for: Determinants and effects of academic engagement in university–industry collaboration: a PLS-SEM approach
Source: Front Psychol. 2026 Apr 22;17:1745917. doi: 10.3389/fpsyg.2026.1745917 (PMC13143539; doi:10.3389/fpsyg.2026.1745917)

Appendix A2


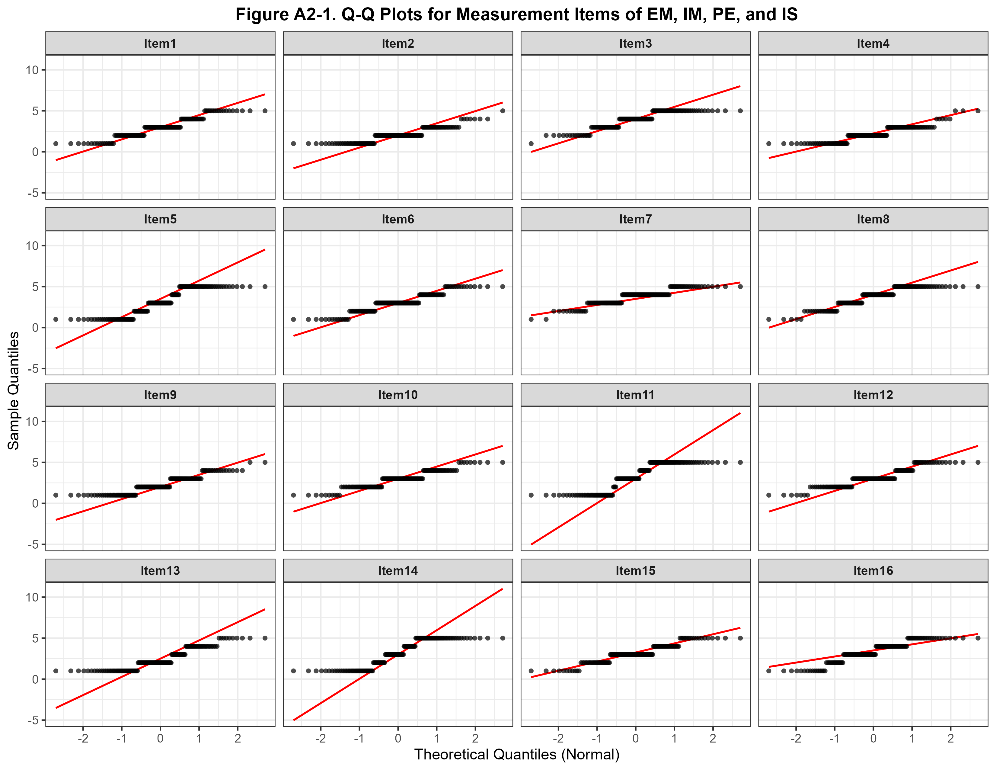


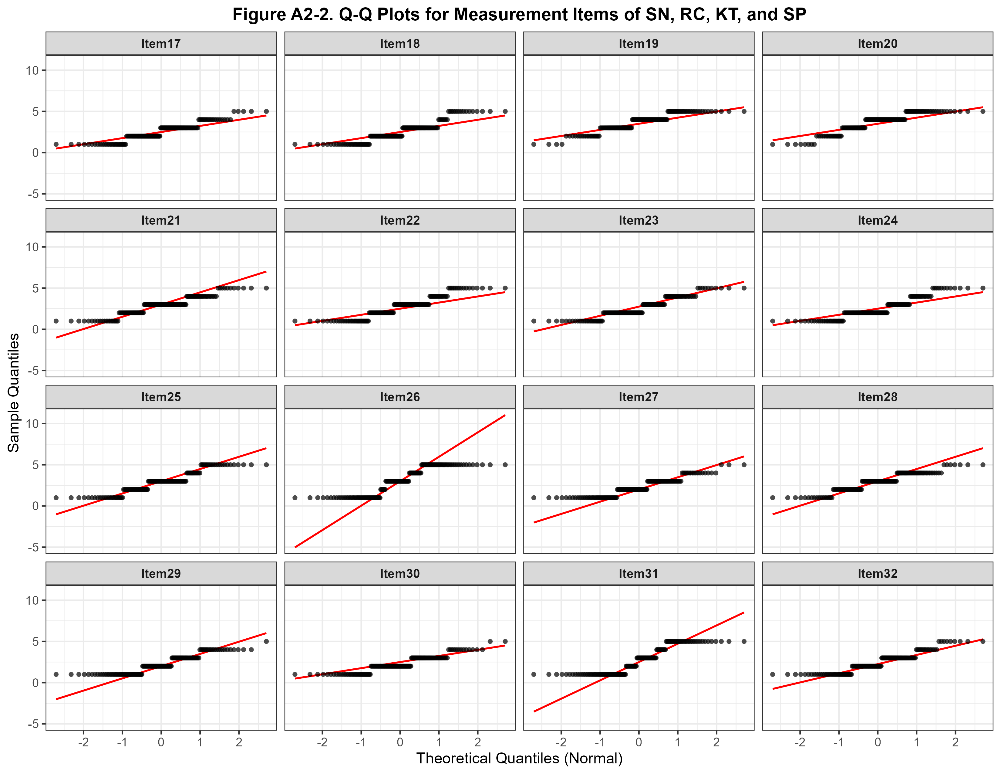

Supplement: Supplementary file 2 [file Supplementary_file_2.docx]
